# The effects of alfentanil on emergence agitation in children under general anesthesia: a meta-analysis of randomized controlled trials

**DOI:** 10.3389/fped.2025.1607279

**Published:** 2025-10-09

**Authors:** Yuanling Xu, Kun Ding, Xuemei Zhao, Yingying Sun

**Affiliations:** Department of Anaesthesiology, Anhui Provincial Children’s Hospital, Hefei, Anhui, China

**Keywords:** emergence agitation, alfentanil, pediatric, general anesthesia, review

## Abstract

**Background:**

Emergence agitation (EA) is characterized by excessive reactivity and sensory impairment that occurs in children after general anesthesia. Alfentanil is a µ-opioid receptor with rapid onset and short duration, widely used in minor surgery. The aim of this meta-analysis is to assess the effect of alfentanil on the incidence of EA in children undergoing general anesthesia.

**Methods:**

PubMed, Cochrane Library, Embase, and Web of Science databases were reviewed to search for related trials published before April 30, 2025. The primary outcome was the incidence of EA. Secondary outcomes included rescue analgesia, postoperative nausea and vomiting (PONV), emergence time, extubation time, and time to discharge from post-anesthesia care unit (PACU).

**Results:**

The study extracted from 5 studies including 532 patients. Compared to saline, alfentanil reduced the incidence of EA in children (RR = 0.54; 95% CI: 0.42–0.70; *P* < 0.01). In addition, alfentanil decreased the use of rescue analgesic (RR = 0.5; 95% CI: 0.38–0.65; *P* < 0.01), did not increase the incidence of PONV (RR = 1.39; 95% CI: 0.67–2.88; *P* = 0.37). According to the GRADE system, the quality of evidence was moderate for the incidence of EA.

**Conclusions:**

Limited available evidence suggests that alfentanil is associated with a lower incidence of EA in children. However, further high-quality studies are needed to verify the effect of alfentanil in preventing the occurrence of EA in children undergoing general anesthesia.

**Systematic Review Registration:**

https://www.crd.york.ac.uk/PROSPERO/view/CRD42023448260, PROSPERO CRD42023448260.

## Introduction

Emergence agitation (EA) is an acute behavioral disturbance occurring in patients during recovery from general anesthesia (1). EA can occur at any age, but it is more common in pediatric populations, with an incidence rate of about 10%–80% (2, 3). Children with EA typically exhibit irritability, uncooperativeness, and inconsolability, often accompanied by crying, moaning, writhing, and kicking. Although EA is usually self-limiting behavior, it can increase the risks of self-injury, wound dehiscence, bleeding, and dislodgment of catheters. Additionally, children with EA have a higher risk of anxiety, apathy, sleep and eating disorders within 30 days postoperatively (4–6).

The occurrence of EA may be attributed to various factors. Potential risk factors for EA include pre-school children, volatile anesthetics, especially sevoflurane, type of surgery such as otolaryngology and ophthalmic surgeries, body temperature in children, pain, and preoperative anxiety in children and parents (7). The precise mechanism of EA remains unclear, and the search for ways to prevent and treat EA is urgent and necessary. Although there is no universal protocol for managing anesthesia in EA, emerging research has demonstrated the effectiveness of pharmacological methods in preventing EA. A meta-analysis found that dexmedetomidine significantly reduced the incidence of EA in children (8). Multiple studies have shown that drugs such as nalbuphine, midazolam, and melatonin can reduce the occurrence of EA in pediatric patients after general anesthesia (9–11). Besides, magnesium sulfate, a non-competitive NMDA receptor antagonist, emerges as a potential preventive agent against EA (12). Similarly, several studies have found that opioids can effectively reduce the incidence of EA in children (13, 14). Alfentanil is a µ-opioid receptor agonist known for its rapid onset, short duration, and has little effect on cardiovascular and respiratory system, commonly used for minor surgeries (15). Studies have shown that the use of alfentanil in adenotonsillectomy can effectively reduce the incidence of EA (16). However, the addition of alfentanil 10 min before the end of strabismus or epiblepharon repair surgery did not significantly improve the incidence of EA in children (17).

Therefore, the aim of this study was to assess the effect of alfentanil on the incidence of agitation during recovery in children under general anesthesia using systematic review and meta-analysis. It also evaluated the impact of alfentanil on secondary outcome measures such as postoperative nausea and vomiting (PONV). The study provided evidence-based medical evidence for optimizing the management strategy of children's recovery period under general anesthesia.

## Methods

This meta-analysis was conducted in accordance with the Preferred Reporting Items for Systematic Reviews and Meta-Analyses (PRISMA) guidelines (18). The study has been registered in PROSPERO (CRD42023448260).

### Search strategy

Two authors independently conducted a literature search in four electronic databases: PubMed, EMBASE, Web of Science, and Cochrane Library, with the search cutoff date set at April 30, 2025. The electronic database search was supplemented by manually searching the reference lists of included articles. The detailed search strategies for each database are available in the Supplementary Material S1.

### Study selection

After searching for and removing duplicates, two additional researchers independently reviewed abstracts and full-text articles. Any discrepancies were resolved through discussion with a third researcher to reach a consensus decision.

### Criteria for study

The inclusion criteria were as follows: (1) children aged between 0 and 18 years; (2) intervention with alfentanil administered intravenously or intranasally, compared with saline or other drugs (e.g., ketamine); (3) evaluate the incidence of EA; (4) randomized controlled trials; (5) elective non-cardiac surgeries under general anesthesia; (6) publications in English. The exclusion criteria were as follows: (1) cardiac surgery; (2) emergency surgery.

### Data extraction

The authors extracted following data from eligible studies: author names, publication year, country of origin, duration of the study, type of surgery, total number of participants, baseline patient characteristics (mean age, gender), details of the intervention and control protocols, outcomes, and details of methodological quality.

### Primary and secondary outcomes

The primary outcome was the incidence of EA. Secondary outcomes included the number of patients requiring rescue analgesia, PONV, extubation time, emergence time, and time to discharge from PACU. For the measurement of EA, the quadruple scale or the PAED scale were mainly used (19, 20). EA was considered present when the PAED score was more significant than 12 or four-point scale score was ≥3.

### Quality and risk of bias assessment

Two independent researchers used the Cochrane Risk of Bias tool to assess the quality based on seven domains (21): random sequence generation, allocation concealment, blinding of participants and personnel, blinding of outcome assessment, incomplete outcome data, selective outcome reporting, and other sources of bias. According to the Cochrane tool's standards, the risk of bias for each domain was marked as low, unclear, or high.

The Grading of Recommendations Assessment, Development and Evaluation (GRADE) system was employed to assess the level of certainty, yielding four distinct results: high, moderate, low, and very low.

### Data analysis

Summary estimates of categorical and continuous variables were presented as risk ratio (RR) and mean difference(MD), respectively, with each effect size accompanied by a 95% confidence interval (CI). Heterogeneity was assessed by *I*^2^ between-study and the thresholds of ≥25%, ≥50%, and ≥75% represented low, moderate, and high heterogeneity, respectively. Due to clinical heterogeneity, a random-effects model was used for the meta-analysis (22). This model provides an appropriate estimate of the average treatment effect when studies are statistically heterogeneous, typically resulting in relatively wider CI and thus more conservative effects. This meta-analysis did not perform publication bias because the Cochrane's Handbook onsiders the test to be too low when fewer than 10 studies were included (23). The analysis was conducted using Cochrane Review Manager version 5.4, with a significance level set at *P* < 0.05.

## Results

### Study selection and study characteristics

**Figure 1:**
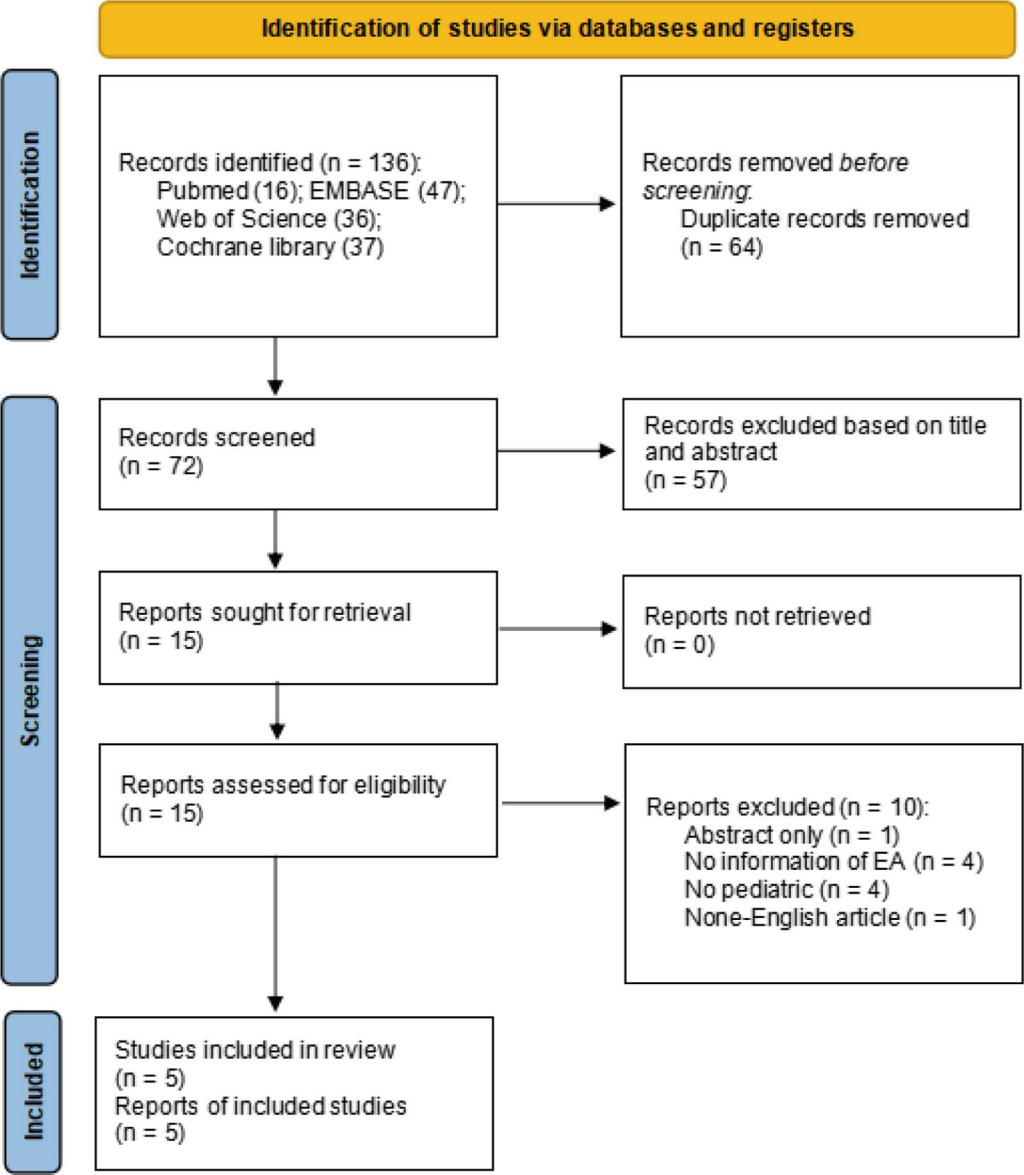
A PRISMA flow diagram of included/excluded studies.

The search identified 136 relevant studies from 4 databases, 64 studies were excluded due to duplication, and 57 were excluded after title and abstract screening. Additionally, 15 articles were eligible to undergo full-text review, of which 10 were excluded for not meeting the inclusion criteria. A total of 5 randomized studies were ultimately included in the meta-analysis (Figure 1). The five studies included were published from 2009 to 2022 (16, 17, 24–26). The sample size of the included trials ranged from 78 to 172, totaling 532 participants. The age of the subjects ranged from 1 to 10 years. The types of surgery performed were tonsillectomy, adenotonsillectomy, urological surgery, ophthalmic surgery, and dental procedures. All patients were anesthetized with sevoflurane inhalation combined with laryngeal mask or endotracheal intubation. The basic characteristics of the included studies are presented in Table 1.

**Table 1:** Characteristics of included studies.

Study. ID	Age (Y)	ASA scale	Surgery type	Anesthesia	Groups	Sample size	Incidence of EA	EA assessment	Outcome
Kim et al. (24)	3–10	I/II	Adenotonsillectomy	Sev, N2O; Intubation	Alfentanil1: 10 μg/kg	32	34%	A four-point score and PAED	ACDF
					Alfentanil2: 20 μg/kg	34	35%		
					NS: volume-matched iv	34	71%		
Bilgen et al. (25)	1–8	I/II	Urological surgery	Sev, N2O Caudal block; LMA	Ketamine: 2 mg/kg	26	3.8%	PAED	ABCE
					Alfentanil: 10 μg/kg	25	36.0%		
					NS: 1 ml intranasal	27	40.7%		
Choi et al. (17)	4–9	I/II	Ophthalmic surgery	Sev; Intubation	Remifentanil: 0.1 μg/kg/min	34	32%	A four-point Score and PAED	ADF
					Remifentanil + single injection alfentanil:5 μg/kg	35	31%		
					NS: volume-matched iv	33	64%		
Zhao et al. (26)	3–6	I	Dental procedure	Sev; LMA	Alfentanil1: 0.2 μg/kg/min	57	22.9%	A four-point score and PAED	ABEF
					Alfentanil2:0.4 μg/kg/min	57	21.1%		
					NS: volume-matched iv	58	48.3%		
Zhang et al. (16)	3–7	I/II	Tonsillectomy alone and adenotonsillectomy	Sev; Intubation	DEX: 0.4 μg/kg	20	25%	A four-point score and PAED	ABCDE
					DEX + Alf1: 10 μg/kg	20	5%		
					DEX + Alf2: 20 μg/kg	20	Never		
					NS: volume-matched iv	20	50%		

A, emergence agitation; B, PONV; C, requiring rescue analgesic; D, extubation time; E, emergence time; F, time to discharge from the PACU; Y, years; DEX, dexmedetomidine; NS, normal saline; LMA, laryngeal mask airway; Sev, sevoflurane.

### Risk of bias

**Figure 2:**
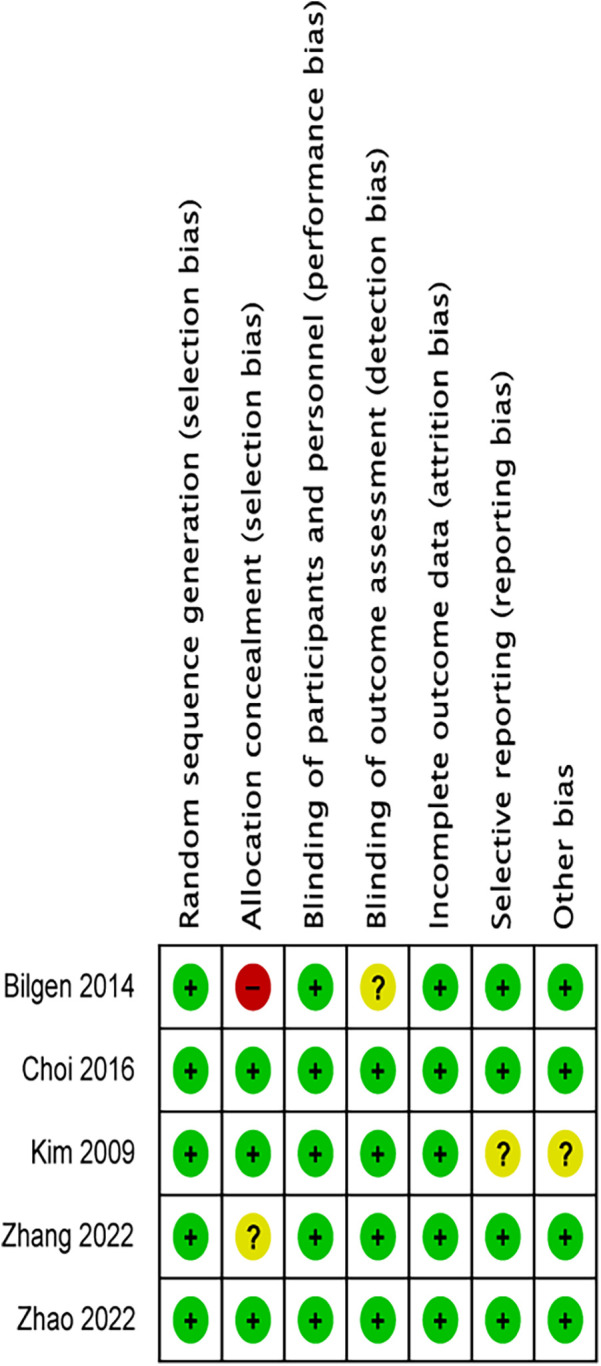
Risk of bias summary.

The article by Kim 2009 using alfentanil at 10 μg/kg is denoted as Kim 2009–1, and the 20 μg/kg group is Kim 2009–2; in the article by Zhao 2022, alfentanil at 0.2 μg/kg/min is labeled as Zhao 2022–1, and 0.4 μg/kg/min as Zhao 2022–2; in the article by Zhang 2022, the 10 μg/kg alfentanil group is Zhang 2022–1, and the 20 μg/kg dose group is Zhang 2022–2. The risk of bias is summarised in Figure 2.

### Meta-analysis of the primary outcome

**Figure 3:**
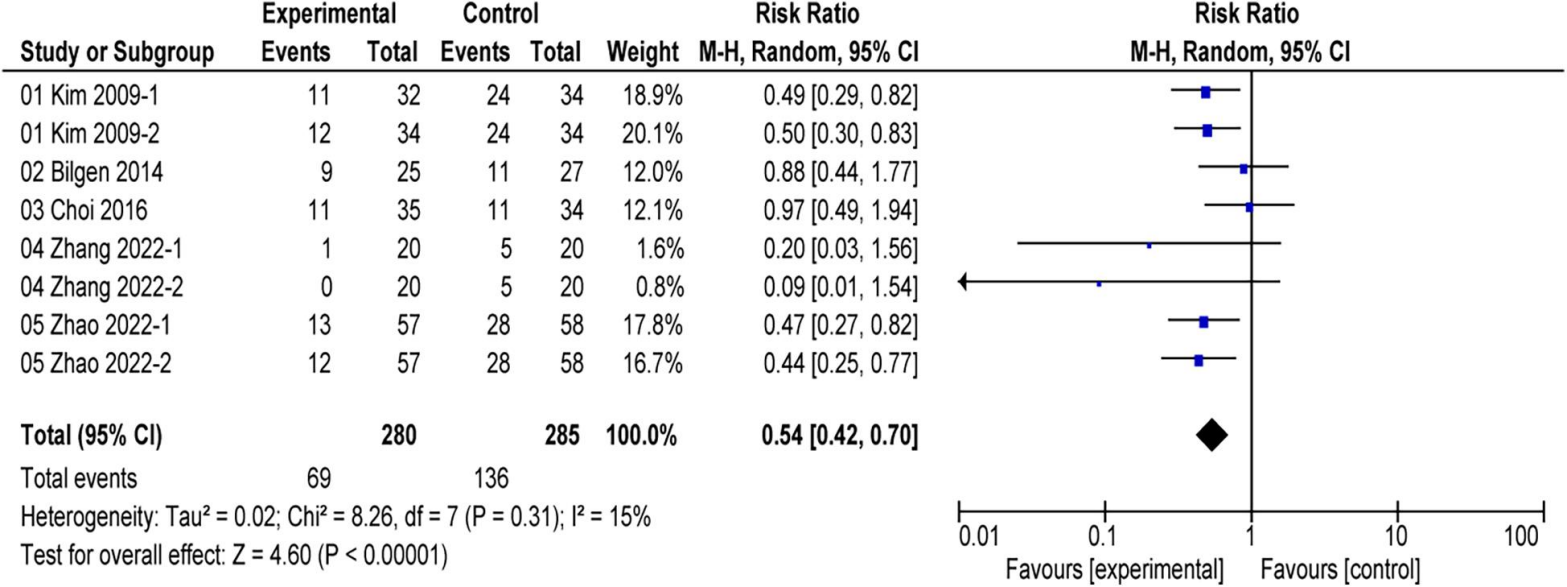
Forest plot of meta-analysis of the incidence of emergence agitation (EA): afentanil vs. Saline.

**Figure 4:**
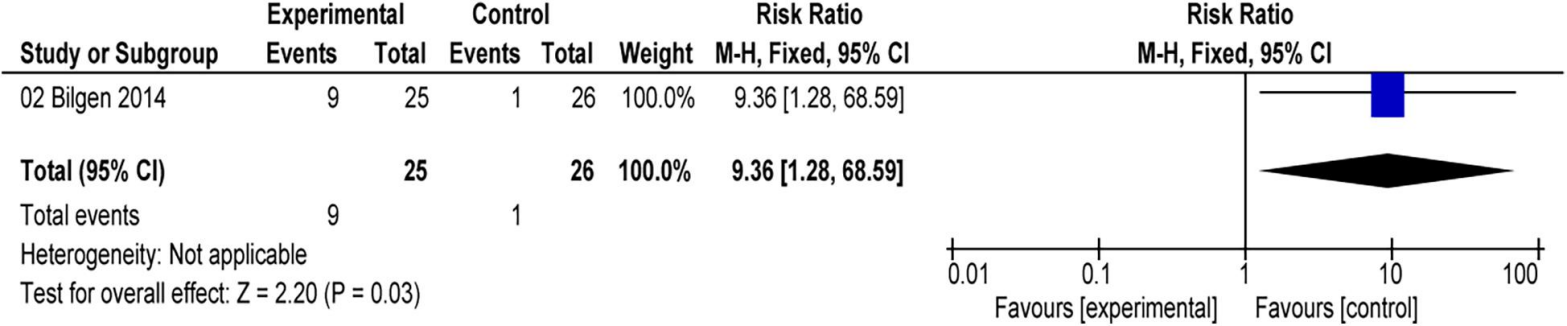
Forest plot of meta-analysis of the incidence of emergence agitation (EA): afentanil vs. ketamine.

All included studies reported the incidence of postoperative EA (16, 17, 24–26). The comprehensive results of the forest plot showed that the incidence of EA was lower with alfentanil as compared with saline group (RR = 0.54; 95% CI: 0.42–0.70; *P* < 0.01; *I*^2^ = 15%) (Figure 3). Additionally, the study by Sevgi compared the effect of alfentanil and ketamine on EA (25), and the meta-analysis showed that the incidence of EA was lower in ketamine group than in alfentanil group (RR = 9.36; 95% CI: 1.28–68.59; *P* = 0.03) (Figure 4).

### Subgroup analysis of the primary outcome

**Figure 5:**
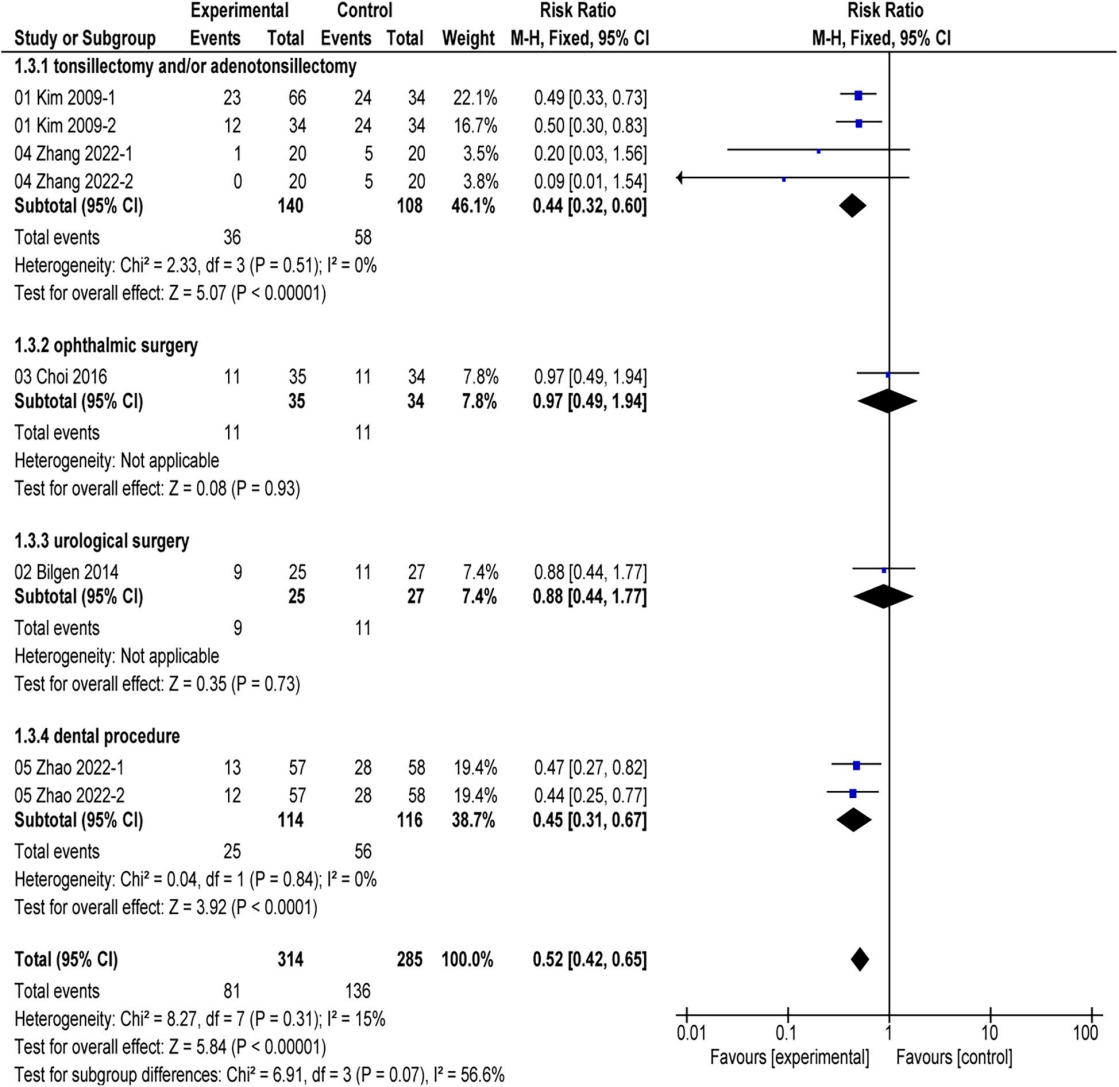
Incidence of EA in different surgeries: alfentanil vs. saline.

**Figure 6:**
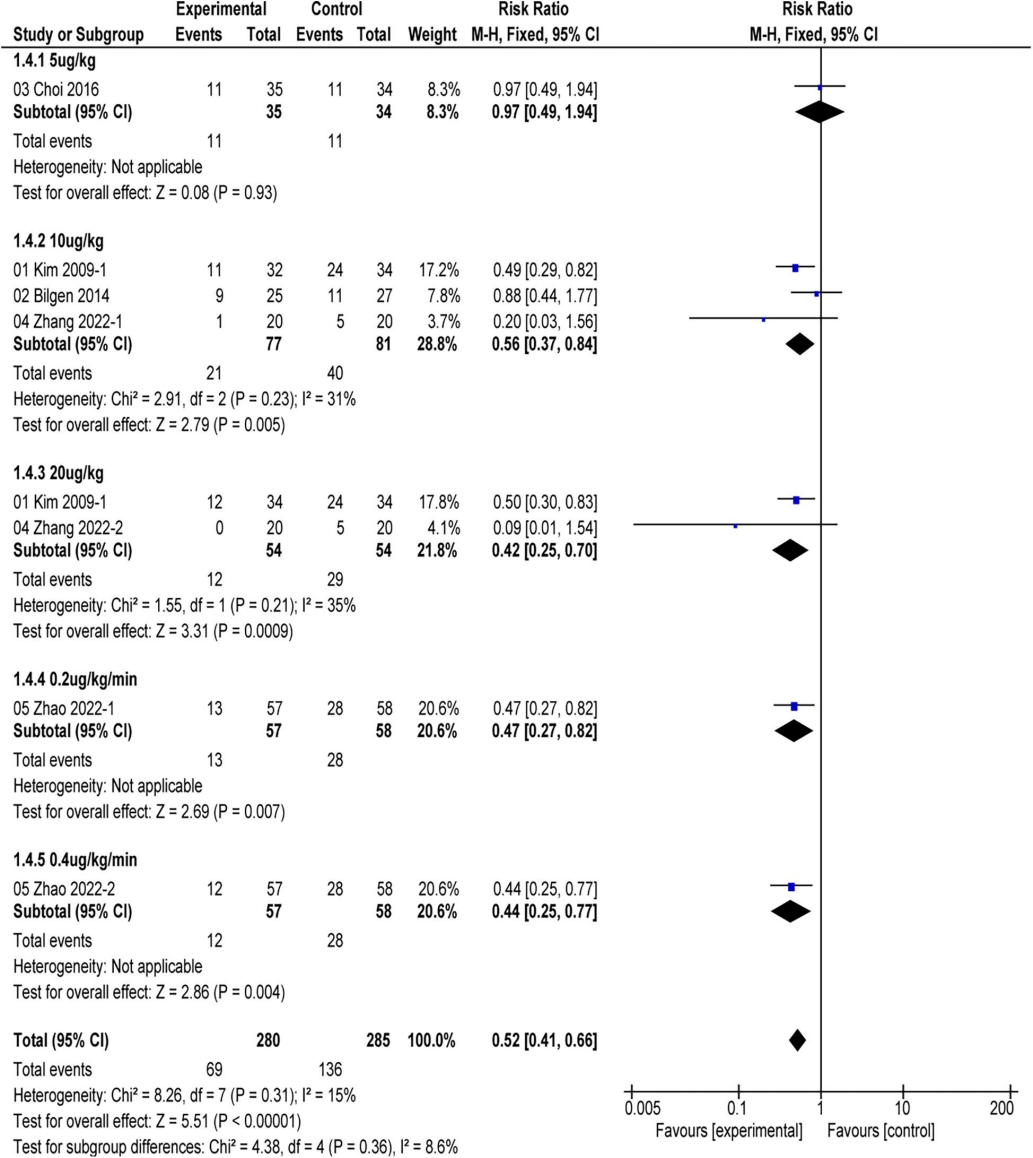
Incidence of EA in different dosages of alfentanil vs. saline.

In the subgroup analysis, two studies (16, 24) indicated that alfentanil compared to saline in tonsillectomy and/or adenoidectomy procedures reduced the incidence of EA (RR = 0.44, 95% CI: 0.32–0.60; *P* < 0.01; *I*^2^ = 0%). Choi's study (17) demonstrated that the combination of remifentanil and alfentanil did not reduce the incidence of EA in ophthalmic surgery compared to remifentanil alone (RR = 0.97; 95% CI: 0.49–1.94; *P* = 0.93) (Figure 5). The use of alfentanil in combination with midazolam did not reduce the incidence of EA in urological surgery compared to midazolam alone (RR = 0.88; 95% CI: 0.44–1.77; *P* = 0.73) (25). However, alfentanil reduced the incidence of EA compared with saline in oral surgery (RR = 0.45; 95% CI: 0.31–0.67; *P* < 0.01; *I*^2^ = 0%) (26). Four studies (16, 24–26) found that the use of alfentanil at doses of 10 µg/kg, 20 µg/kg, 0.2 µg/kg/min, and 0.4 µg/kg/min all reduced the incidence of EA (*P* < 0.01) (Figure 6).

### Meta-analysis of the secondary outcomes

Three studies documented the incidence of PONV (16, 25, 26), postoperative rescue analgesics (16, 24, 25), extubation time (16, 17, 24), emergence time (16, 25, 26) and the time discharge from PACU (17, 24, 26). Compared to the saline group, alfentanil significantly reduce the percentage of children requiring postoperative rescue analgesics (RR = 0.5; 95% CI: 0.38 to 0.65; *P* < 0.01, *I*^2^ = 0%). However, the administration of alfentanil has been linked to prolonged extubation time (MD = 0.85; 95% CI: 0.18 to 1.51; *P* = 0.01, *I*^2^ = 79%), emergence time (MD = 2.60; 95% CI: 1.69 to 3.52; *P* < 0.01, *I*^2^ = 84%), and the time discharge from PACU(MD = 1.63; 95% CI: 0.87 to 2.38; *P* < 0.01, *I*^2^ = 85%). Moreover, alfentanil has no significant effect on PONV (RR = 1.39; 95% CI: 0.67–2.88; *P* = 0.37, *I*^2^ = 0%) (Table 2). In comparison with other drug (25), alfentanil significantly increased the number of children requiring postoperative rescue analgesics compared to ketamine (RR = 9.36; 95% CI: 1.28 to 68.59; *P* = 0.03), while reducing emergence time compared to ketamine significantly (MD = −5.00; 95% CI: −9.28 to −0.72; *P* = 0.02). There was also no significant difference in PONV between the two drugs (RR = 0.78; 95% CI: 0.32 to 1.93; *P* = 0.59) (Table 3).

**Table 2:** Secondary outcomes of Alfentanil vs. Saline.

Outcome	Number of studies	Number of participants	RR or MD	95%CI	Heterogeneity/*I*^2^	*P* value
PONV	3	284	1.39	0.67 to 2.88	0%	0.37
Requiring rescue analgesic	3	212	0.5	0.38 to 0.65	0%	<0.01
Extubation time	3	229	0.85	0.18 to 1.51	79%	0.01
Emergence time	3	238	2.60	1.69 to 3.52	84%	<0.01
Time to discharge from PACU	3	295	1.63	0.87 to 2.38	85%	<0.01

RR, risk ratio; MD, mean difference; PONV, postoperative nausea and vomiting; PACU, post-anesthesia care unit.

**Table 3:** Secondary outcomes of Alfentanil vs. Ketamine.

Outcome	RR or MD	95%CI	*P* value
PONV	0.78	0.32 to 1.93	0.59
Requiring rescue analgesic	9.36	1.28 to 68.59	0.03
Emergence time	−5.00	−9.28 to −0.72	0.02

RR, risk ratio; MD, mean difference; PONV, postoperative nausea and vomiting.

### GRADE assessment

All included studies were randomized trials, and the assessors were blinded. The risk of bias for some outcomes was graded as “serious” due to the high risk for selection bais in one of the included studies. For outcomes with moderate or substantial heterogeneity, we evaluated the level of inconsistency as “serious (30% ≤ *I*^2^ < 60%)” or “very serious (*I*^2^ > 60%)”. According to the GRADE system, the quality of evidence was moderate for the incidence of EA, PONV and requiring rescue analgesic, while low for Extubation time and Time to discharge from PACU. And the quality of evidence was very low for emergance time (Table 4).

**Table 4:** Grading of recommendations assessment, development, and evaluation (GRADE) evidence profile (Alfentanil vs. Saline).

Outcomes	Study design	Number of studies	Relative effect (95% CI)	*I* ^2^	Risk of bias	Certainty assessment				Certainty (GRADE)
						Inconsistency	Inconsistency	Imprecision	Imprecision	
The incidence of EA	RCT	5	0.54 (0.42 to 0.7)	15%	Serious^a^	not serious	Not serious	Not serious	None	⊕⊕⊕○ Moderate
PONV	RCT	3	1.39 (0.67 to 2.88)	0%	Serious^a^	not serious	Not serious	Not serious	None	⊕⊕⊕○ Moderate
Requiring rescue analgesic	RCT	3	0.5 (0.38 to 0.65)	0%	Serious^a^	not serious	Not serious	Not serious	None	⊕⊕⊕○ Moderate
Extubation time	RCT	3	0.85 (0.18 to 1.51)	79%	Not serious	very serious^b^	Not serious	Not serious	None	⊕⊕○○ Low
Emergance time	RCT	3	2.60 (1.69 to 3.52)	84%	Serious^a^	very serious^b^	Not serious	Not serious	None	⊕○○○ Very Low
Time to discharge from PACU	RCT	3	1.63 (0.87 to 2.38)	85%	No serious	very serious^b^	Not serious	Not serious	None	⊕⊕○○ Low

EA, emergence agitation; RR, risk ratio; PONV, postoperative nausea and vomiting; PACU, post-anesthesia care unit.

^a^ One of the included studies was rated high risk.

^b^ The heterogeneity was moderate (*I*^2^ > 60%).

## Discussion

This meta-analysis indicates that compared with saline, alfentanil can significantly reduce the incidence of EA in children after general anesthesia, decrease postoperative rescue analgesics use, and has no effect on PONV, but it prolongs extubation time, emergence time, and the time of discharge from PACU.

EA is one of the most common complications of pediatric anesthesia, characterized by excitement, restlessness, and other unusual behaviors, such as crying, kicking, inconsolability, and non-cooperation (27, 28). Although the etiology of EA in children remains unclear, certain risk factors for EA generally considered are as follows:preschool age, preoperative anxiety, postoperative pain, and inhalational anesthetic (7, 29). Drug therapy isone of the effective methods. Studies have found that *α*-receptor agonists such as dexmedetomidine, midazolam, opioid receptor agonists such as nalbuphine, and alfentanil can reduce the incidence of EA in children (30, 31). Consistent with Tan's study (32), this meta-analysis shows that alfentanil significantly reduced the incidence of EA compared to saline. In subgroup analyses of different surgical types on the incidence of EA, the use of alfentanil in ophthalmic and urological surgeries showed no significant impact compared to saline. We speculate that it may be related to the combination of alfentanil with other anesthetic drugs in these two articles. In Bilgen's study, all patients received permedication with oral midazolam 0.5 mg/kg before anesthesia induction. Oral midazolam has been proven to effectively reduce the incidence of EA in children in multiple studies (33, 34). In Choi's study, both the alfentanil group and the control group received continuous infusion of remifentanil during surgery. Remifentanil has also been shown to reduce the incidence of EA in children after general anesthesia (35, 36).

In subgroup analyses of different doses of alfentanil, we found that alfentanil at doses of 10 µg/kg, 20 µg/kg, 0.2 µg/kg/min, and 0.4 µg/kg/min all reduced the incidence of EA compared to saline, while alfentanil at 5 µg/kg showed no effect on the incidence of EA compared to saline. However, only 3 and 2 of the 5 included articles recorded the effects of 10 µg/kg and 20 µg/kg of alfentanil on EA, while only 1 article recorded 5 ug/kg, 0.2 µg/kg/min and 0.4 µg/kg/min. Due to the limited research available, it still cannot determine the optimal and minimum dose of alfentanil for preventing EA in children after general anesthesia. Meanwhile, the effect of using alfentanil at different times on EA remains unclear. Thus, The optimal level of alfentanil to control EA should be determined in future studies.

Postoperative acute pain is recognized as an important risk factor for EA, and inadequate analgesia can lead to it, as described in the PAED scale (37). Three studies reported the need for postoperative rescue analgesics. The results of our meta-analysis showed that the use of alfentanil significantly reduced the use of postoperative rescue analgesics compared with the use of control group. Therefore, the effect of pain on postoperative agitation could not be completely excluded in this study. Alfentanil is a synthetic, short-acting µ-opioid agonist that can effectively alleviate pain and may prevent the occurrence of EA (32). It is worth noting that pediatric patients, due to their limited language expression abilities, often exhibit pain-related defensive movements (such as kicking and resisting treatment). These behaviors can be difficult to distinguish from the irrational agitation caused by disorientation during the anesthesia emergence period. This phenotypic overlap may lead to an overestimation of the proportion of non-painful agitation during emergence from anesthesia (6, 38). Face, Legs, Activity, Cry, Consolability (FLACC) scores is a common tool to assess the degree of pain in infants and young children (39). It scores patients based on five behavioral indicators, helping healthcare professionals quantify the pain level and develop appropriate interventions. Cai's team proposed a method to differentiate postoperative pain from emergence agitation using both the FLACC and PAED (Pediatric Assessment of Emergence Delirium) scales (40). For children with a FLACC score of ≥4, acetaminophen or fentanyl is administered to mitigate the potential impact of pain on the assessment of postoperative emergence agitation (40).

Moreover, this meta-analysis shows that alfentanil prolong extubation time, emergence time, and the time discharge from PACU. However, it must be emphasized that the results of these three time indicators all exhibit a high heterogeneity (*I*^2^: 79%; 84%; 85%). We used sensitivity analysis to eliminate the included literature one by one, but the heterogeneity was still high. This suggests that heterogeneity may be caused by a combination of methodological or clinical differences between studies. In Zhang's study, the use of dexmedetomidine may have affected the extubation time and emergence time (16). In addition, the definition of emergence time varies in different studies, including spontaneous eye opening time, time from discontinuation of sevoflurane until the children acted on command, and first eye opening or crying (16, 25, 26). In terms of the time to discharge from PACU, although the Aldrete score was used in all studies, there were differences in the threshold (9 or 10 points) and implementation details (whether parents accompanied them) that affected the consistency of the decision to leave the room (16, 17, 24).

This meta-analysis still has limitations. First, the sample size is relatively small, which consequently compromises the overall precision of the findings. Future research necessitates the conduct of additional high-quality multicenter randomized controlled trials. Second, the literature included in this study was only published in English, which might lead to certain publication bias. Third, the literature included in this meta-analysis used the PAED scale and the 4-point scale, and we did not separate these two scales for statistics. This is because we only analyzed the incidence of EA, but not the severity of EA. Future studies are needed to further analyze the severity of EA to provide a more precise rationale for clinical treatment. Fourth, whether different opioids affect the incidence of EA requires further study. Although study showed that an additional single dose of alfentanil before the end of surgery did not significantly reduce the incidence of EA when remifentanil is continuously infused (17). However, it remains to be studied whether the incidence of EA is different with continuous infusion of alfentanil and other opioids. Fifth, the heterogeneity of some of the secondary outcomes was high. In addition, the GRADE approach assessed the quality of evidence for these outcomes as low and very low quality. This indicates the uncertainty in the results.

## Conclusion

Compared to saline, alfentanil reduces the incidence of EA and the requirement for rescue analgesics in children undergoing general anesthesia, without increasing the incidence of PONV. However, it prolongs extubation time, emergence time, and time to discharge from PACU. Practical decisions should weigh core benefits against potential costs. For children at high risk of EA, a controlled recovery delay might be a reasonable choice to exchange for stable awakening. For low-risk or cases requiring rapid turnover, the need for medication should be individually evaluated and the dosing strategy optimized. Given the limitations of small sample size and low-quality evidence, future high-quality research is necessary to provide further effective estimates of the effect of alfentanil in preventing EA in pediatric surgical patients.

## Data Availability

The raw data supporting the conclusions of this article will be made available by the authors, without undue reservation.
