# Supplementary material for: The effects of alfentanil on emergence agitation in children under general anesthesia: a meta-analysis of randomized controlled trials
Source: Front Pediatr. 2025 Oct 9;13:1607279. doi: 10.3389/fped.2025.1607279 (PMC12545061; doi:10.3389/fped.2025.1607279)
Supplement: Supplementary file 1 [file Datasheet1.docx]

Supplementary Material

**1. Search strategy**

**Pubmed 16**

#1 Alfentanil[MeSH Terms]

#2 (((((((((Alfentanyl[Title/Abstract]) OR (Alfentanil Hydrochloride[Title/Abstract])) OR (R-39209[Title/Abstract])) OR (R 39209[Title/Abstract])) OR (R39209[Title/Abstract])) OR (Alfenta[Title/Abstract])) OR (Limifen[Title/Abstract])) OR (Rapifen[Title/Abstract])) OR (Fanaxal[Title/Abstract]))

#3 #1 OR #2

#4 emergence delirium[MeSH Terms]

#5 ((emergence*[Title/Abstract] OR postoperative [Title/Abstract] OR postanesthetic [Title/Abstract]) AND (agitat*[Title/Abstract] OR delirium [Title/Abstract] OR confus*[Title/Abstract] OR "behavioral change*"[Title/Abstract] OR excitement [Title/Abstract]))

#6 #4 OR #5

#7 (randomized controlled trial [Publication Type] OR controlled clinical trial [Publication Type] OR randomized [Title/Abstract] OR placebo [Title/Abstract] OR clinical trials as topic [MeSH: noexp] OR randomly [Title/Abstract] OR trial [Title]) NOT (animals [MeSH Terms] NOT humans [MeSH Terms])

#8 #3 AND #6 AND #7

**Cochrane 37**

#1 MeSH descriptor: [Alfentanil] explode all trees

#2 Alfentan* OR R-39209 OR R 39209 OR R39209 OR Alfenta OR Limifen OR Rapifen OR Fanaxal

#3 #1 OR #2

#4 MeSH descriptor: [Emergence Delirium] explode all trees

#5 (emergence* OR postoperative OR postanesthetic) and (agitat* OR delirium OR confus* OR behavioral change* OR excitement)

#6 #4 OR #5

#7 #3 AND #6

**Embase 47**

#1 'alfentanil'/exp

#2 alfentanyl:ab,ti OR 'alfentanil hydrochloride':ab,ti OR 'r 39209':ab,ti OR r39209:ab,ti OR alfenta:ab,ti OR limifen:ab,ti OR rapifen:ab,ti OR fanaxal:ab,ti

#3 #1 OR #2

#4 'emergence delirium'/exp

#5 (emergence*:ti,ab OR postoperative:ti,ab OR postanesthetic:ti,ab) AND (agitat*:ti,ab OR delirium:ti,ab OR confus*:ti,ab OR 'behavioral change':ti,ab OR excitement:ti,ab)

#6 #4 OR #5

#7 'crossover procedure':de OR 'double-blind procedure':de OR 'randomized controlled trial':de OR 'single-blind procedure':de OR random*:de,ab,ti OR factorial*:de,ab,ti OR crossover*:de,ab,ti OR ((cross NEXT/1 over*):de,ab,ti) OR placebo*:de,ab,ti OR ((doubl* NEAR/1 blind*):de,ab,ti) OR ((singl* NEAR/1 blind*):de,ab,ti) OR assign*:de,ab,ti OR allocat*:de,ab,ti OR volunteer*:de,ab,ti

#8 #3 AND #6 AND #7

**Web of science 36**

#1 TS=（Alfentanil OR Alfentanyl OR Alfentanil Hydrochloride OR R-39209 OR R 39209 OR R39209 OR Alfenta OR Limifen OR Rapifen OR Fanaxal）

#2 TS= （（emergence* or postoperative OR postanesthetic）SAME （agitat* OR delirium OR confus* OR "behavioral change*" OR excitement ））

#3 TS= clinical trial* OR TS=research design or TS=comparative stud* OR TS=evaluation stud* OR TS=controlled trial* OR TS=follow-up stud* OR TS=prospective stud* OR TS=random* OR TS=placebo* OR TS=（single blind*）OR TS=（double blind*）

#4 #1 AND #2 AND #3
